# Hand specific representations in language comprehension

**DOI:** 10.3389/fnhum.2014.00360

**Published:** 2014-06-03

**Authors:** Claire Moody-Triantis, Gina F. Humphreys, Silvia P. Gennari

**Affiliations:** ^1^Department of Psychology, University of YorkYork, UK; ^2^Neuroscience and Aphasia Research Unit, School of Psychological Sciences, University of ManchesterManchester, UK

**Keywords:** language comprehension, action execution, action representations, premotor cortex, left hand, right hand, mirror neurons

## Abstract

Theories of embodied cognition argue that language comprehension involves sensory-motor re-enactments of the actions described. However, the degree of specificity of these re-enactments as well as the relationship between action and language remains a matter of debate. Here we investigate these issues by examining how hand-specific information (left or right hand) is recruited in language comprehension and action execution. An fMRI study tested self-reported right-handed participants in two separate tasks that were designed to be as similar as possible to increase sensitivity of the comparison across task: an action execution go/no-go task where participants performed right or left hand actions, and a language task where participants read sentences describing the *same* left or right handed actions as in the execution task. We found that language-induced activity did not match the hand-specific patterns of activity found for action execution in primary somatosensory and motor cortex, but it overlapped with pre-motor and parietal regions associated with action planning. Within these pre-motor regions, both right hand actions and sentences elicited stronger activity than left hand actions and sentences—a dominant hand effect. Importantly, both dorsal and ventral sections of the left pre-central gyrus were recruited by both tasks, suggesting different action features being recruited. These results suggest that (a) language comprehension elicits motor representations that are hand-specific and akin to multimodal action plans, rather than full action re-enactments; and (b) language comprehension and action execution share schematic hand-specific representations that are richer for the dominant hand, and thus linked to previous motor experience.

## Introduction

Theories of embodied cognition argue that language understanding implies partially simulating or re-enacting the actions being described and thus involves brain regions that are recruited in the execution of those actions (Jeannerod, 2001; Glenberg and Kaschak, 2002; Barsalou et al., 2003; Gallese and Lakoff, 2005; Barsalou, 2008). Indeed, it has been found that body part specific regions of the motor system are activated when reading language describing actions (Hauk et al., 2004; Buccino et al., 2005; Pulvermuller, 2005; Tettamanti et al., 2005) and they do so to an effort specific degree (Moody and Gennari, 2010), suggesting that language recruits detailed action representations that would also be required for the execution of the same specific action.

However, the nature of the representations that are shared between action and language remains unclear, in particular, their level of specificity, i.e., to what extent do we re-enact the execution of an action described by language? Indeed, both primary motor and pre-motor regions have been associated with language comprehension and these contrasting findings imply different levels of specificity in the representations elicited by language: if primary motor regions are recruited during language comprehension, comprehenders can be thought to more closely re-enact the action described as if they were performing it, because these regions are directly connected to the spinal cord and musculature (Dum and Strick, 1996). Alternatively, if pre-motor and parietal regions are recruited, comprehenders may activate more schematic action plans that do not involve execution aspects *per se*, since pre-motor regions are typically associated with planning (Cisek et al., 2003).

The view that language may involve highly specific action representations is consistent with fMRI language studies that have reported the recruitment of primary motor regions (Hauk et al., 2004; Rüschemeyer et al., 2007; Kemmerer et al., 2008; Kemmerer and Gonzalez-Castillo, 2010) and with TMS studies showing that stimulation of primary motor cortex during language comprehension modulates body-part specific motor evoked potentials (Oliveri et al., 2004; Buccino et al., 2005; Candidi et al., 2010). In contrast, the view that language involves more schematic action representations is supported by many language studies showing the recruitment of planning-related pre-motor and parietal regions, rather than primary motor regions (Noppeney et al., 2005; Aziz-Zadeh et al., 2006; Moody and Gennari, 2010; Willems et al., 2010; Meteyard et al., 2012).

To shed light on this issue, we conducted an fMRI study directly comparing action execution and language comprehension. The tasks were designed to be as similar as possible to increase the sensitivity of the comparison. Every participant performed an action execution and a language comprehension task. We focused on hand-specificity, i.e., whether the action is performed, or described as performed, with the left or the right hand. Importantly, the actions included in the execution task held a one-to-one correspondence with the content of the sentences read in the language task. Thus, participants executed left and right hand button presses in the execution tasks and correspondingly read sentences describing left or right hand button presses in the language task, albeit in different syntactic forms. In both tasks, participants were required to match a visual cue (e.g., L, R) referring to a left or right hand action with the execution of the action itself or the content of the sentence, thus keeping participants focused on the directionality of the stimuli.

This design has the potential of providing more homogeneous activations and more precise and sensitive comparisons across conditions than previous studies. First, the linguistic stimuli utilized refer to the same action, instead of classing together different verbs (e.g., *grasp, touch, give*), which often have different senses and syntactic properties. Second, the linguistic meanings targeted in the experiment had a one-to-one correspondence with the actions executed in the execution task, unlike previous studies comparing meaningless actions (e.g., finger movements) with semantically complex verbs (e.g., *grasp*) (Aziz-Zadeh et al., 2006). Finally, the execution task preceded the language task to encourage imagery during the language task, thus increasing the chances to detect potentially weak activity in primary motor regions.

Importantly, the focus on hand specificity provides simple ways to distinguish between primary-motor and premotor regions, say, in comparison to body-part manipulations, because the activation patterns for left and right hand actions within primary motor and pre-motor cortices is relatively well understood. Indeed, primary motor cortex has long been thought to play an important role in the control of limbs on the contralateral side of the body (Tanji et al., 1988; Dassonville et al., 1997; Aziz-Zadeh et al., 2002; Cisek et al., 2003). Thus, executing, observing or imagining a right-hand movement would recruit more neurons and stronger activity in the left primary motor cortex, and vise-versa. In contrast, activity in pre-motor regions responds to both right and left hand actions both in cell recording and fMRI studies (Tanji et al., 1988; Kermadi et al., 2000; Cisek et al., 2003; Hanakawa et al., 2006; Horenstein et al., 2009), although they may respond to different degrees (see below). This is due to the fact that the pre-motor cortex houses more schematic representations responsible for planning rather than executing actions, and thus, are less directly linked to the spinal cord (Rizzolatti and Luppino, 2001).

Therefore, we predicted that if language comprehension involves hand-specific representations, the pattern associated with either execution or planning of left and right hand actions in primary motor or premotor areas should also be observed in language comprehension. Specifically, if language recruits schematic planning representations only, then a similar pattern of activity across language comprehension and planning should be found in pre-motor areas, but if linguistic representations are more detailed in execution content, language comprehension should match the execution-specific activity pattern in primary motor regions, i.e., a contralateral pattern.

## Materials and methods

### Participants

Eighteen participants were recruited for the experiment, all reported to be right-handed native English speakers with no known neurological disorders, and to use the right hand in daily and sport activities (14 female, 4 male; mean age 21, age range 19–23 years).

### Material

In the execution tasks, visual letter cues were used to elicit button presses that could include one or two fingers (e.g., LX, RX, LL, RR). In the language comprehension task, all sentences were written in the first person narrative (e.g., *I am pressing…*.) to encourage the activation of the participant's own motor experience during language comprehension. Each sentence described left/right hand button presses using either one or two fingers. In total 160 action sentences were presented. To encourage participants to process the sentence meaning and to maintain their attention, the phrasing of the sentence was varied, for example, when describing one button press with the left hand participants could read one of 4 different sentences (see Table 1). The length in characters of the sentences varied from 27 to 47 (mean length 37.25), however to ensure that the sentences were all matched across conditions, the same structure was used in the left and right conditions, with the words *right* and *left* varying accordingly. Therefore, psycholinguistic variables such as length and frequency should not influence the results.

**Table 1:** **Sentence stimuli**.

**Hand action**	**Sentence**
Right: two fingers	*I'm pressing both buttons with my right fingers*
	*I'm pushing two buttons on the right*
	*I'm pushing two right buttons*
	*On the right, I'm pressing two buttons*
Right: one finger	*I'm pressing the button with my right finger*
	*I'm pushing one button on the right*
	*I'm pushing one right button*
	*On the right, I'm pressing one button*
Left: two fingers	*I'm pressing both buttons with my left fingers*
	*I'm pushing two buttons on the left*
	*I'm pushing two left buttons*
	*On the left, I'm pressing two buttons*
Left: one finger	*I'm pressing the button with my left finger*
	*I'm pushing one button on the left*
	*I'm pushing one left button*
	*On the left, I'm pressing one button*

### Task procedure and design

Ethical approval for the study was obtained from Ethics committee at the York Neuroimaging Centre, where the study was carried out. Before the scanning session, participants were familiarized with the letter patterns to be used in the execution task. They practiced this task until they felt confident. They then practiced the subsequent language task, which used the same cues but required different motor responses. All participants performed the execution task before the language task.

#### Action execution task

A go-no-go task was used to elicit button presses. During the experiment, participants held one button box in each hand resting on their lap in a comfortable position. Each button box had two buttons and participants were instructed to rest their index and middle fingers on the buttons of the boxes during the experiment. Visual stimuli were projected through a mirror fixed to the head coil. The go/no-go cues were pairs of letters in red uppercase 50 pt text. In total 200 action stimuli were presented, 160 go trials and 40 no-go trials. The go trials instructed participants to press either one (RX, LX) or two buttons (RR, LL) using either the right or the left hand as quickly as possible (there were 40 trials per cue). During practice, participant learned to match each letter of the visual cue onto each of the four buttons (and fingers), so that RX indicated one button press with the right middle finger, RR indicated pressing both buttons simultaneously (middle and index finger) and so on. The no-go trials instructed participants to refrain from pressing a button (either XR or XL, i.e., an initial X meant no response at all). Visual cues lasted for 500 ms and were then replace by *HH*, which stayed on the screen until the next cue. Cues from different conditions (left/right) using one or two fingers were intermixed in an event-related design following optimal stimulus order (the probability of each condition following any other condition was constant) and random inter-trial times obtained by a schedule optimizing algorithm (http://surfer.nmr.mgh.harvard.edu/optseq/). Therefore participants could not predict the upcoming stimulus and had to plan each trial. Inter-trial interval varied in duration from 2 to 26 s (average 5.8 s). The task lasted 960 s in total.

#### Language comprehension task

Participants remained in the scanner in the same position and holding the same button boxes as in the previous task. Participants were presented with 160 sentences in white 30 pt text (on black background) each lasting 2000 ms and were asked to read the sentences for meaning. Table 1 exemplifies the different formats in which sentences were presented (10 cases of each example). After each sentence presentation, a sequence of 37 X's were presented (which constitute the average character length of all sentential stimuli) until next stimulus sentence appeared. To keep participants' attention on the sentential content, 34 catch trials (also lasting 2000 ms) were also included in the design (21.25% of trials). As in action execution, an event-related design was used where trial types were intermixed in such a way that the probability of each trial type (sentence conditions plus catch trials) following any other type was constant, and therefore trial types could not be predicted (the order of trials and inter-trial times were calculated with the same schedule optimizing algorithm as above). Inter-trial intervals ranged from 2 to 26 s (average 4.96 s). Catch trials asked participants about the sentence content using the same cues that were used in the execution task, e.g., *RR?* Participants had to indicate whether the meaning of the previously read sentence corresponded to the cue (meaning judgment task). To respond to this question, they had to use a left hand button press (index finger for *yes* and middle finger for *no*). For example, participants may read *I'm pushing two buttons on the right*, and after a few seconds (corresponding to the variable inter-trial time), they may be presented with *RR?*, in which case, the correct answer is *yes* (a left index finger button press). In order to perform well on this task participants had to read the sentences carefully for their hand-specific action meaning, and therefore it ensured that participants maintained their attention throughout the experiment.

### Data collection parameters

A 3T GE Signa Exite MRI scanner was used to collect both high-resolution structural images and functional images. Functional images were obtained using a gradient-echo EPI sequence (TR 2000 ms, TE 50 ms, flip angle 90°, matrix 64 × 64, field of view 24 cm) with 38 axial slices of thickness 3.0 mm. The resulting voxel size was 3.75 × 3.75 × 3 cm. Note that our TE specification is near those considered optimal for detecting signal in primary motor cortex (Fera et al., 2004). Functional images excluded the cerebellum and in some participants inferior portions of the temporal lobe. A T1 flair image was also obtained in order to facilitate the registration between the high-resolution structural and functional data.

### Data analysis

Both first level and higher-level analyses were carried out for the language and the action task separately using FEAT (FMRI Expert Analysis Tool) Version 5.91, part of FSL (FMRIB's Software Library, www.fmrib.ox.ac.uk/fsl). We have followed the standard order of processes built into the FSL FEAT analysis. Pre-processing steps included brain extraction, slice-timing correction, motion correction (Jenkinson et al., 2002), spatial smoothing using a Gaussian kernel of FWHM 8 mm and high-pass temporal filtering (Gaussian-weighted least-squares straight line fitting, with sigma = 25.0 s). Time-series analysis was carried out using FILM with local autocorrelation correction (Woolrich et al., 2001). A boxcar model of the timing of events was created involving the onset and length of each stimulus event, which was then convolved with a hemodynamic response (gamma) function. For both action and language data the events were modeled at the onset of the stimulus presentation with action trials lasting 500 ms and language trials lasting 2000 ms. For the language task, the catch trials were modeled separately to partial out the participant's motor responses but were excluded from any statistical average or comparison of the language data. No-go trials in the execution task were also modeled out and not analyzed further.

Several contrasts were run at the individual level between the different conditions in the execution and the language task. For both the execution and the language data, all actions or sentences together (irrespective of hand) were compared to rest to identify all action or all language regions, and right and left hand actions or sentences were also compared against one another to find those areas that were significantly more involved in performing or reading about left or right hand actions (R > L, L > R). Individual level analyses were then entered into high-level mixed-effect modeling built into FSL, taking into account both variance and parameter estimates from individual-level results. All higher-level analyses reported below were carried out using FLAME (FMRIB's Local Analysis of Mixed Effects (Woolrich et al., 2004) within the right or left hemisphere to increase statistical power. Z (Gaussuanised T/F) statistic images were thresholded using a Gaussian Random Field-theory (GRF)-based maximum height with a (corrected) significance threshold of *p* = 0.05 (Worsley et al., 1992). For convenience, we will refer to this correction method, *GRF-based correction*.

#### Region of interest analyses in hand- and execution-specific regions

**Figure 1:**
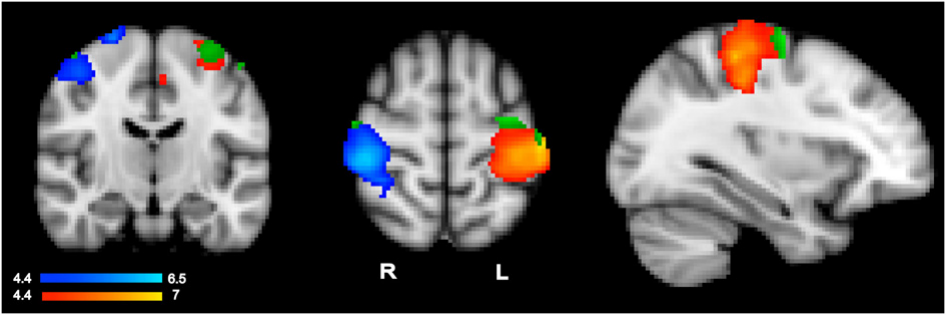
**Results from the action execution task showing the contralateral pattern of activation specifically responding to left hand actions (in blue, left hand > right hand contrast) and right hand actions (in red, right hand > left hand) (whole brain GRF-based correction, *p* = 0.05)**. Significant language comprehension activity responding to all sentence types within each execution region is shown in green.

To evaluate whether language activity within primary motor regions showed the same pattern as that of action execution, we used execution-specific activity to identify regions for further analyses of hand-specific language activity. To isolate hand-specific execution regions that would not include common planning regions, we used execution activity resulting from contrasting left-hand and right-hand actions, i.e., the contrasts R > L and L > R, obtained with GRF-based correction at *p* = 0.05. Subtracting left from right and right from left action performance should cancel out any general planning activity that is shared across hands, thus identifying execution specific activity, which should show the typical contralateral pattern. Indeed, simply comparing left-hand or right-hand execution relative to rest may still include regions that are common to both hands, and thus likely to reflect common planning regions, because these general contrasts only identify voxels active for one hand irrespective of the other hand. These contrasts yielded as expected, the contralateral pattern shown in in Figure 1 in the blue-to-cyan and red-to-yellow scales. Within these hand- and execution-specific contralateral ROI masks, we then ran a high-level analysis (GRF-based correction, *p* = 0.05) for the language data irrespective of hand, i.e., the contrast all sentences vs. rest, to establish whether language comprehension activated these hand-specific execution regions. This yielded significant language activity (irrespective of hand) shown in green in Figure 1. The average percent signal change within the significant cluster resulting from this high-level analysis was then extracted for each participant using FSL tools. *T*-tests (with subjects as random factor) were then used to determine whether there was any difference between left-hand and right-hand sentences.

#### Region of interest analyses in non-hand-specific regions

**Figure 2:**
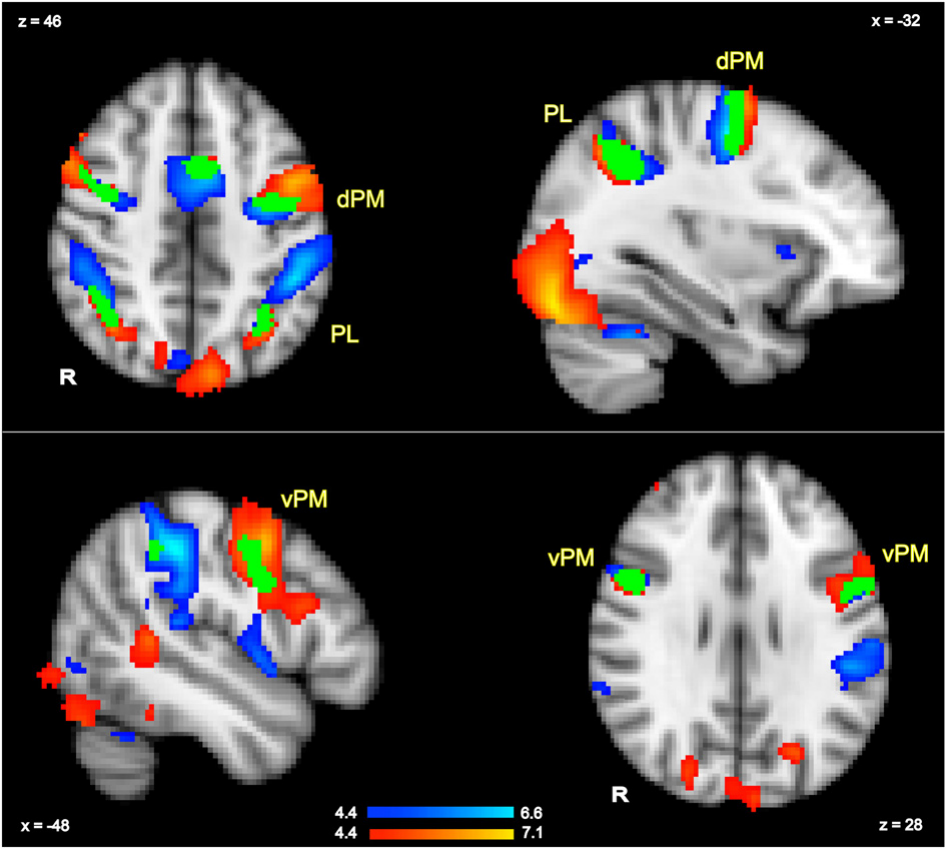
**Action execution activity (in blue) and language comprehension activity (in red) in response to all actions and all sentence stimuli compared to rest (whole brain GRF-based correction, *p* = 0.05)**. The regions in which language and execution activity overlapped (conjunction) are shown in green and are labeled as dorsal pre-motor (dPM), ventral pre-motor (vPM) and parietal lobe (PL).

To isolate regions that were sensitive to all hand actions irrespective of hand and thus were likely to include activations in planning regions, we contrasted all actions relative to rest (GRF-based correction, *p* = 0.05). The corresponding contrast was also conducted in the language task to identify all regions involved in language comprehension irrespective of hand (GRF-based correction, *p* = 0.05). By multiplying these execution and language comprehension results, we localized several clusters that were significantly active in both tasks, and thus indicated overlapping regions across tasks. This is equivalent to a conjunction analyses as previously referred to in the literature (Nichols et al., 2005). These overlapping clusters thus acted as functional localizers for the regions targeted for further analysis of more specific contrasts (Poldrack, 2007). In particular, to establish whether there were hand-specific activations within these overlapping regions, we extracted the percent signal changes for each hand relative to rest for each participant in each of the main overlapping clusters shown in Figure 2. These values were then analyzed with paired *t*-tests (with subjects as random factor) to examine whether either in action planning or in language comprehension, there was stronger activity for a specific hand, and more generally, to examine whether a similar pattern of activity was shown for planning and language, as hypothesized.

## Results

### Behavioral data

#### Execution task

The time taken to perform the instructed action and the number of errors made were measured.

***Reaction times.*** Trials containing errors or responses longer than 3 standard deviations from the mean were excluded from the reaction times analyses. These exclusions constituted about 3.40% of the total data. We found that participants responded faster with the right hand (mean = 615.8 ms) than the left hand (mean = 630.2 ms) [*t*_(18)_ = 2.77, *p* = 0.01], thus providing supporting evidence that our participants were indeed right-handed.

***Accuracy.*** A response was classed as an error if participant either failed to make a response or responded using the wrong hand. On average participants made an error on 3.06% of action trials, although there was not reliable difference between left and right hand actions (Wilcoxon Signed-Rank test: *z* = −0.637, *p* > 0.05). The numbers of errors were also calculated on no-go trials, with errors being defined as those no-go trials where an action was incorrectly performed. On average, errors on no-go trials were relatively low and were made 2.5% of the time. Furthermore, almost all errors (94%) were consistent with the directional letter in the cue (i.e., if the cue was XR the right button was most likely to be erroneously pressed, and vise-versa).

#### Language task

Due to experimenter error, no responses were recorded from one participant. For the remaining 17 participants, on average participants responded correctly on 90.7% of the question trials and the mean reaction time for the responses was 2605 ms, as measured from the presentation of the cue (e.g., *RR?*).

### Overall functional activations for action execution and language comprehension

#### Action representations in hand- and execution-specific regions

As anticipated from previous research, hand-specific action execution (left > right and right > left) elicited stronger responses in the contralateral hemisphere (GRF-based correction, *p* = 0.05) (Figure 1). The strongest activity was centered around the post central gyrus and extended into the central sulcus and pre-central gyrus (left hemisphere peak: −40, −26, 54; right hemisphere peak: 42, 4–30, 58). The corresponding corrected analysis for the language comprehension data contrasting one hand relative to the other however did not elicit any significant response. To make sure that stringent correction level did not miss hand-specific language activity, we conducted further ROI analyses within the contralateral execution clusters, as described in Region of Interest Analyses in Hand- and Execution-specific Regions and reported below.

#### Actions representations in non-hand-specific (planning) regions

The contrast of all actions relative to rest (GRF-based correction, *p* = 0.05) revealed several brain regions that were commonly activated by the execution task irrespective of hand. These included premotor and parietal regions, as well as other regions. Peak activations for the left-hemisphere are listed in Table 2, and the overall pattern of execution activity is shown in the blue-to-cyan scale in Figure 2. The contrast of all sentences relative to rest also revealed several brain regions that included parietal, pre-motor, posterior temporal and inferior frontal regions (GRF-based correction, *p* = 0.05). Peak activations for the left-hemisphere are listed in Table 2, and the overall language activity is shown in the yellow-to-red scale in Figure 2. The multiplication of the activity elicited by each of these tasks indicated regions that were significantly activated for both action execution/planning and language (conjunction), as shown in green in Figure 2. These common activations suggest that common neural representations were recruited for both execution/planning and language comprehension. The overlapping regions were located in the middle frontal gyrus/dorsal pre-central gyrus, superior parietal lobule/angular gyrus, and ventral pre-central gyrus and were larger in the left than the right hemisphere. Because these regions were associated with more than one anatomical label according to the Harvard-Oxford Cortical Structural Atlas, henceforth we refer to them as dorsal or ventral pre-motor regions (dPM, vPM) and parietal lobe regions (PL). The centers of gravity of these regions are listed in Table 2.

**Table 2:** **Peak activations for each task and center of gravity for overlapping regions**.

**Tasks**	**Anatomical label**	**MNI *x*,*y*,*z***	**z**	**Voxels in cluster**
Action execution	Post central gyrus	−4, −50, 74	4.87	60
	Precentral gyrus	−32, −10, 52	6.51	415
		−60, 4, 14	5.58	59
		−54, 6, 28	4.57	57
		−48, −4, 42	5.45	15
	Cingulate gyrus/SMA	−8, 2, 42	5.98	391
	SMA	2, −6, 70		14
	Supramarginal gyrus	−46, −36, 42	6.64	1047
	Superior frontal gyrus	−20, −6, 72	5.33	22
	Opercular cortex	−48, 0, 0	5.3	29
	Lateral occipital cortex	−40, −74, 0	5.23	97
		−22, −70, 32	4.68	14
Language comprehension	Pre−central gyrus	−44, 0, 42	6.54	926
	Inferior frontal gyrus	−56, 14, 18	5.5	110
	Middle temporal gyrus	−54, −48, 6	6	191
	SMA	−2, −2, 70	5.73	207
	Precuneous	0, −68, 60	5.89	566
	Superior parietal lobule	−32, −60, 42	6.22	166
	Fusiform cortex	−44, −44, −20	6.14	110
	Lateral occipital cortex	−24, −72, 30	5.28	28
Execution + language areas (center of gravity)	Precentral gyrus/middle frontal gyrus (dorsal premotor—dPM)	−34, −7, 55		419
	Precentral Gyrus (ventral pre-motor—vPM)	−50, 0, 33		351
	Parietal lobe	−34, −52, 40		410
	Supplementary motor area	−1, 3, 52		324

*Coordinates are given for the left hemisphere, which were analogous to those in the right hemisphere. Cluster sizes are given for the GRF-based corrected images at a threshold of z = 4.5*.

### Region of interests

#### Hand- and execution-specific regions

Hand specific language activity was assessed in two steps (see section Region of Interest Analyses in Hand- and Execution-specific Regions) because direct contrast between left- and right-hand sentences did not show any significant voxel in a high-level analysis masked by the hand- and execution-specific ROIs of Figure 1. We first conducted a high-level analysis within hand specific execution ROIs to detect any language activity irrespective of hand (all sentences vs. rest). This analysis revealed significant clusters shown in green in Figure 1. The clusters were located in the superior portion of the pre-central gyrus (left hemisphere peak: −32, −10, 64). Within these clusters, we then evaluated hand-specific activity by extracting the percent signal change for left and right hand sentences vs. rest for each participant and for each of the left and right hemisphere clusters. *T*-tests comparing left vs. right hand sentence activity within these clusters revealed no significant difference (*p* > 0.4). The hand specific pattern of data as seen in action performance is therefore not seen when comprehending hand specific action language within these execution areas.

#### Non-hand specific (planning) regions

**Figure 3:**
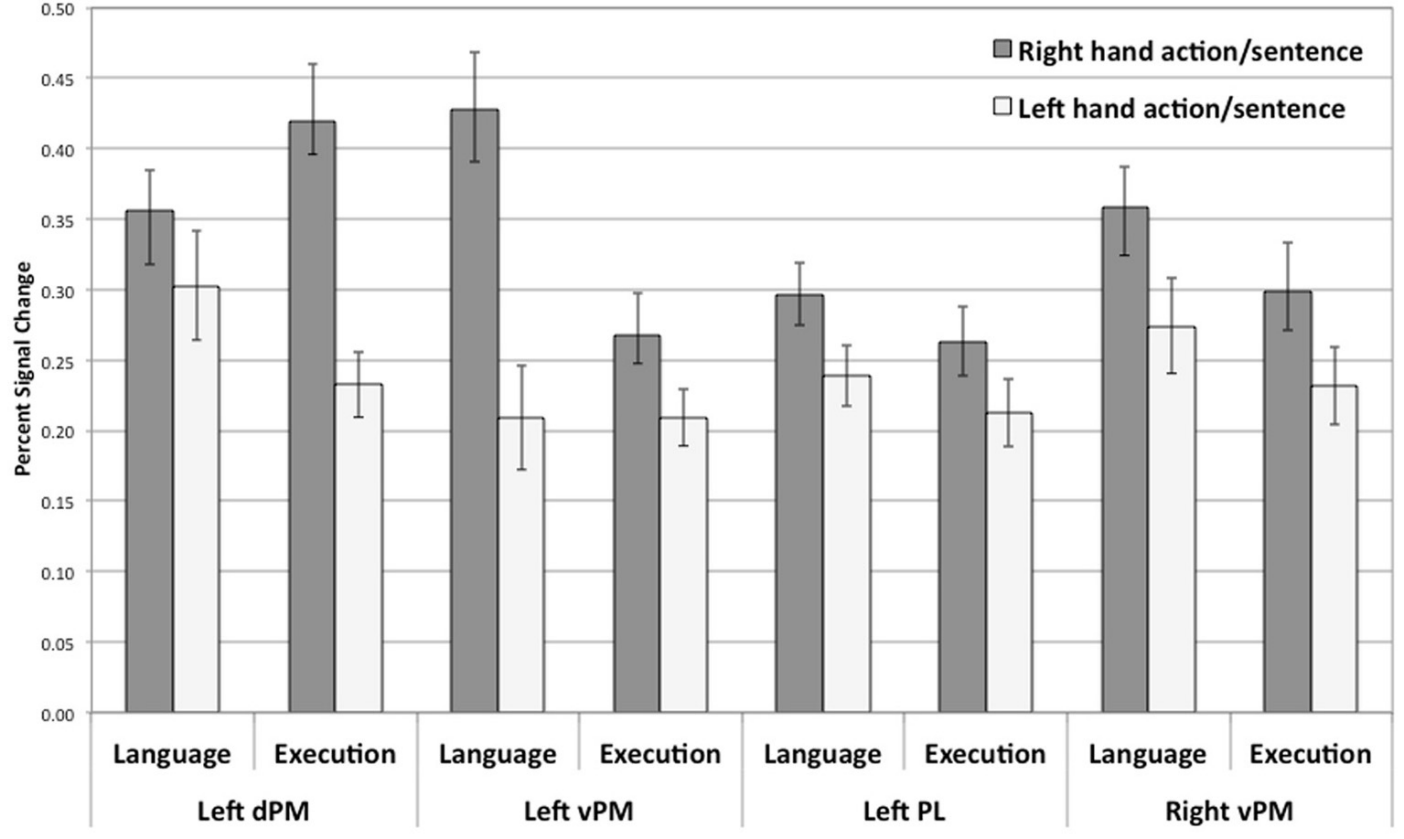
**Percent signal change for right or left hand actions and right or left hand sentences within regions of overlap between action execution and language comprehension (see Figure 2)**. All comparisons are significant at *p* < 0.02. Error bars represent standard error.

To examine whether a similar pattern of activity was shown for planning and language within the regions that were significantly activated in both tasks, as hypothesized, for each of the identified common regions of activation for the language and execution tasks (see above and Figure 2), we contrasted right and left hand actions or sentences for each of the hemispheres. The overall pattern of results is summarized in Figure 3. For all the common clusters of activation in the left hemisphere, we found a parallel pattern of activation across action execution and language comprehension. As shown in Figure 3, right-hand actions or sentences elicited stronger activity than left-hand actions or sentences [dPM—language activity: *t*_(17)_ = 2.71, *p* < 0.02; dPM—execution activity: *t*_(17)_ = 5.98, *p* = 0.0001; PL—language activity: *t*_(17)_ = 3.42, *p* < 0.003; PL—execution activity: *t*_(17)_ = 2.46, *p* < 0.03; vPM—language activity: *t*_(17)_ = 2.53, *p* < 0.01; vPM—execution activity: *t*_(17)_ = 2.53, *p* < 0.02]. For the common clusters of activation in the right hemisphere, the pattern of results was numerically similar to that in the left hemisphere, with right-hand actions or sentences also eliciting a stronger response than left hand actions or sentences. However, only the vPM cluster showed statistically significant results for execution and language [language activity: *t*_(17)_ = 3.61, *p* < 0.002; execution activity: *t*_(17)_ = 2.96, *p* < 0.009], with all other right-hemisphere regions not reaching significance (*p* > 0.05). Note that these results, and particularly those in pre-motor regions, could not be due to eye-movements during reading, which we could not control for: First, left vs. right sentences were identical except for one word, and thus are likely to elicit similar eye-movements. Therefore, the differences in hand-specific activity cannot be due to more or less eye-movement in one condition relative to other. Second, the coordinate range typically associated with the frontal-eye field (Paus, 1996; Swallow et al., 2003) do not correspond to those reported here, consistent with the fact that this region is anterior to the hand area. Finally, the execution task, with which language activity overlapped, only involved central fixation, and therefore, cannot be due to eye-movements.

Overall, these results suggest that hand-specific effects are found in regions of common activity for action planning and language comprehension in left pre-motor and parietal regions and right pre-motor regions. Because these regions were active for the execution of either hand action and were not located in primary motor regions, they reflect more schematic representations associated with planning, rather than muscle control. Therefore, action execution/planning and language comprehension appear to recruit some aspects of these more schematic representations. Interestingly, both language comprehension and action execution show hand specific effects characterized by stronger responses for the right hand than for those of the left hand, suggesting a dominant hand effect, since our participants reported to be right-handed. We will discuss this specific effect below.

## Discussion

This study aimed to investigate the nature of the representations that are recruited by hand-specific information during language comprehension, and to assess the extent to which we simulate the actions that we read about by comparing language activity to motor-related activity elicited by similar tasks. Participants were asked to perform left and right hand button presses and read sentences that described the same left and right hand button presses. Hand-specific activity for the language task was then assessed within the primary motor hand-specific contralateral regions where execution and language activity overlapped. We predicted that if we nearly accurately re-enact the actions we read about, hand-specific contralateral activity should occur in execution-specific areas such as primary motor cortex for language comprehension in the same way that it does in action execution. This prediction was not supported. Although there was some significant language activity in the superior portion of the pre-central gyrus, a contralateral pattern of activity for hand-specific actions or any sensitivity to hand-specificity was not seen in language as it was in action execution (section Hand- and Execution-specific Regions). This suggests that language comprehension does not show sensitivity to hand-specificity within these execution areas, and therefore that the hand-specific information that was required for language comprehension was not represented within execution areas.

We also predicted that if hand-specific information is represented in a more schematic and general way during language comprehension, then those areas that are responsible for action planning (including the premotor and parietal cortex) would display equivalent activation patterns in the action execution and language task for left and right hand actions. This prediction was assessed in those regions of the premotor and parietal cortex that were activated during action execution *and* language comprehension irrespective of hand, i.e., these regions were significantly active for both left and right hand action or sentences [section Actions Representations in Non-hand-specific (Planning) Regions], but we further examined whether there was any hand-specific differences in the amplitude of this activation [section Non-hand Specific (Planning) Regions]. We found that there was more activity for right-hand actions and right-hand sentences than left ones in most of the pre-motor and parietal regions examined within the left hemisphere (a dominant hand effect) as well as in pre-motor areas of the right hemisphere. This indicates a similar pattern of activation across language comprehension and action execution/planning in pre-motor regions, as predicted. Together these results provide support for embodied cognition and suggest that language recruits detailed hand-specific action representations that are nevertheless one-step removed from re-enacting the execution of the action itself. In other words, language comprehension does not fully activate all action components that are required for the performance of that action. Instead, only more schematic action representations that are stored in areas responsible for action planning are recruited for language.

The dominant hand effect, i.e., that right-hand actions or sentences elicited more activity than left-hand ones in pre-motor regions, is consistent with previous studies suggesting that motor representations in language comprehension and action observation are modulated by motor experience (e.g., Buccino et al., 2004; Calvo-Merino et al., 2005; Beilock et al., 2008). Indeed, language studies have shown that right handers and left handers activate pre-motor cortex to a different degree in different hemispheres (Willems et al., 2010), and activity in pre-motor regions describing hockey actions correlates with different degrees of hockey experience in the dominant hemisphere (Beilock et al., 2008). In action observation studies, more activity is also seen in pre-motor areas for observing human compared to non-human actions (Buccino et al., 2004), biomechanically performable actions compared to non-performable actions (Costantini et al., 2005; Candidi et al., 2008) or those actions that a participant is expert, rather than inexperienced in performing (Calvo-Merino et al., 2005; Haslinger et al., 2005; Cross et al., 2006; Kiefer et al., 2007; Beilock et al., 2008). This suggests that increased experience results in the establishment of a more elaborate action representation leading to stronger activations in action execution, observation, and language comprehension.

Our results are consistent with much of the literature on pre-motor cortex showing that unlike primary motor regions, ventral and dorsal premotor regions play a variety of a cognitive functions supporting not only action planning, e.g., via the formation of visuo-motor associations, but also perceptual analysis, serial prediction and attentional functions (Johnson et al., 1996; Boussaoud, 2001; Picard and Strick, 2001; Simon et al., 2002; Schubotz and von Cramon, 2003; Cisek and Kalaska, 2004; Chouinard et al., 2005). In particular, this research has proposed functional differentiations between dorsal and ventral portions of the premotor cortex (e.g., Schubotz and von Cramon, 2003). In this respect, our results suggest common representations for execution/planning and language comprehension in these two premotor regions, as we found a more dorsal pre-motor cluster in the left hemisphere and another cluster more ventral and bilateral (Figure 2). Although both left hemisphere clusters are located in the proximity of previously reported hand-related motor and language activity, which indeed have been reported to be located either more dorsally or ventrally (see summary of coordinates in Kemmerer and Gonzalez-Castillo, 2010), the fact that two distinct clusters were fund here suggests different roles for these regions. More dorsal aspects of the pre-motor cortex are implicated in spatial attention and specifically, the use of current or expected sensory features of the environment relative to the body (Boussaoud, 2001; Schubotz and von Cramon, 2003), which is consistent with the attention to directionality required in both our tasks relative to the body. Therefore, it is possible that different aspects of the action representation are distributed across the pre-motor cortex, one cluster linked to spatial features and another to motor plans or schemas.

More importantly for the purpose of our study, our results have implications for theories of embodied cognition as applied to language. Although we cannot exclude the possibility that other more sensitive methods or more targeted designs may reveal language sensitivity in primary motor regions, the same experimental conditions that elicited significant effects in pre-motor regions were not sufficient to detect hand-sensitive activity in primary motor regions. Thus, the comprehension of hand action sentences does not seem to involve action representations that are specifically recruited for left or right hand executions in contralateral hemispheres, even when imagery was encouraged by the order and similarity of the execution and language tasks. This suggests that those regions of primary motor cortex directly linked to the spinal cord are not activated by language and language-elicited imagery in similar conditions to those that activate pre-motor regions. This contrasts with previous fMRI and TMS reports, which may have been tapping into planning components and did not distinguish between effector-specific plans and executions. In TMS studies in particular, it is very likely that stimulation of primary motor cortex will stimulate pre-motor cortex too, due to strong interconnections between the two (Chouinard et al., 2003). Therefore, language does not appear to elicit simulations of the action described *as if we were performing the action*, but rather *as if we had the intention or idea of performing the action*.

Nevertheless, we do find stronger activity for the dominant right hand bilaterally in the pre-central gyrus, and in other left pre-motor and parietal regions. According to previous findings, this suggests that action plans or schemas in these regions activate richer representations for the dominant hand, and in this respect, they are hand-specific representations, i.e., they include information as to whether the action would be executed with the left or the right hand. This is particularly revealing because previous language studies have suggested that hand-action representations are body specific, i.e., right and left handers display opposing activation patterns across the hemispheres in premotor regions, with right handers showing more activity in the right hemisphere than the left hemisphere and vice-versa (Willems et al., 2010). Here, we go a step further and show that these pre-motor representations are not only body-specific but also hand-specific. Even more, if the rich experience associated with the dominant hand is indeed responsible for stronger activity, our results suggests that hand dominance is not only represented on the dominant hemisphere but also bilaterally in the pre-central gyrus, suggesting shared functions across the hemispheres.

These observations are consistent with the fact that mirror neurons have primarily been reported in pre-motor regions, rather than primary motor ones, and are considered multimodal, often integrating visual, somatosensory and motor information (e.g., Rizzolatti et al., 2002; Gallese and Lakoff, 2005). It is thus conceivable that language may also activate them, particularly in a task where attention to hand effector and directionality is required. However, these partial re-enactments only support or contribute to language comprehension, as other regions were also recruited for language comprehension but not action execution, most notably, the left inferior frontal gyrus and the posterior temporal lobe (see Figure 2). These two regions have been consistently implicated in many lesion and imaging studies of language processing (Jefferies and Lambon Ralph, 2006; Tyler and Marslen-Wilson, 2008; Humphreys and Gennari, 2014), suggesting that their role is critical to language comprehension. Therefore, our study demonstrates those aspects of the language network where action representations are shared with action planning.

Nevertheless, the cognitive role of mirror-like activity in the brain still remains to be fully understood. Recent findings suggest that mirror-like responses can also be found in primary motor cortex, and that canonical mirror responses can also be found in the hippocampus, SMA and medial frontal regions (Tkach et al., 2007; Lepage et al., 2008; Mukamel et al., 2010). These same regions also display cells with opposite pattern of excitation and inhibition to those observed during action execution or observation, suggesting a role for both integration and differentiation of representations across the brain. Complex activity patterns of neural assemblies across the brain have already been studied in detail by researchers interested in the control of behavior, for which attention and working memory (the need to maintain a goal in memory through complex sequences of actions) are key cognitive processes (e.g., Fuster, 2001). This sort of systemic approach, where temporally integrated activity patterns are investigated across a large network, is likely to provide critical clues for understanding emergent cognitive processes.

## Conclusion

The present results suggest that within the constraints and assumptions of fMRI research, we don't appear to re-enact the actions that we read about in all the same brain areas that are required for action execution. Only very particular action representations are recruited by language—those involved in more abstract stages of action planning in pre-motor cortex. Nevertheless, the representations that are stored in these planning regions are highly specific in that they contain hand-specific information. This is therefore consistent with embodied theories of language proposing that language understanding involves the partial re-enactment of the action described, including hand-specific representations, but we do not accurately re-enact the action as such throughout the motor system. Language understanding is therefore somewhat removed from action execution as it relies upon higher-level cognitive regions.

### Conflict of interest statement

The authors declare that the research was conducted in the absence of any commercial or financial relationships that could be construed as a potential conflict of interest.
